# Acute necrotizing encephalopathy with H1N1 virus infection in a child

**DOI:** 10.1590/0037-8682-0641-2020

**Published:** 2021-03-08

**Authors:** Berhan Pirimoglu, Halil Keskin, Huseyin Tan

**Affiliations:** 1 Ataturk University, Medical Faculty, Department of Radiology, Erzurum, Turkey.; 2 Ataturk University, Medical Faculty, Department of Pediatric Intensive Care Unit, Erzurum, Turkey.; 3 Ataturk University, Medical Faculty, Department of Pediatric Neurology, Erzurum, Turkey.

**FIGURE 1:**
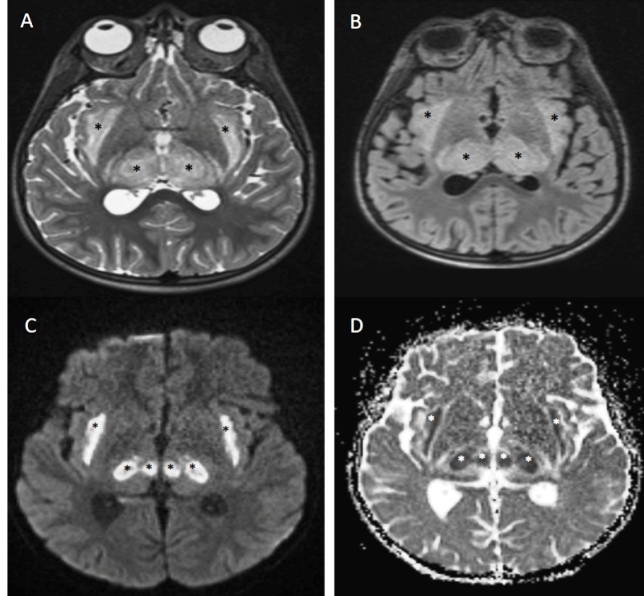
**(A)** Axial T2-weighted image and **(B)** axial FLAIR image show the symmetrical hyperintense expansile lesions in the bilateral thalami and external capsules (black asterisks). **(C)** Axial b-1000 image (black asterisk) and **(D)** axial ADC diffusion image (white asterisk) show the symmetrical diffusion restriction in the bilateral thalami and external capsules.

**FIGURE 2:**
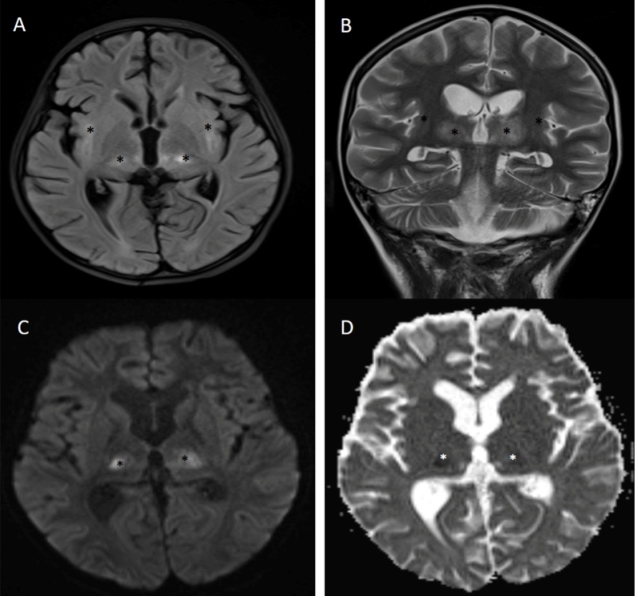
**(A)** Axial FLAIR image and **(B)** coronal T2-weighted image show the decreased symmetrical hyperintense lesions in the bilateral thalami and external capsules (black asterisks). **(C)** Axial b-1000 image (black asterisk) and **(D)**, axial ADC diffusion image (white asterisk) show the regression of symmetrical diffusion restriction pattern in the bilateral thalami

A 7-year-old man presented to our radiology unit with high fever, generalized tonic-clonic type seizures, and neurological deterioration requiring intensive care. Cerebrospinal fluid analysis showed increased protein (94 mg/dL) and normal glucose (41 mg/dL) levels. An initial brain magnetic resonance imaging (MRI) revealed symmetrical and expansile hyperintense lesions in the bilateral thalami and external capsules (Figure 1A and 1B). Restricted diffusion patterns of the involved brain regions were detected (Figure 1C and 1D). These radiological imaging findings suggested acute viral encephalitis, compatible with acute necrotizing encephalopathy (ANEC). Real-time polymerase chain reaction test performed with samples from nasopharyngeal swabs was positive for influenza A virus (H1N1). Therefore, ANEC-associated H1N1 virus infection was diagnosed. The patient underwent antiviral, anticonvulsant, and anti-inflammatory therapy. Fifteen days after therapy, a follow-up brain MRI revealed a decrease in signal intensity in the extent of lesions involving the bilateral thalami and external capsules with decreases in diffusion restriction patterns (Figure 2).

ANEC is an unusual type of encephalopathy characterized by multiple bilateral brain lesions, involving the thalami, putamina, internal and external capsules, cerebellar white matter, and brainstem. It usually develops secondary to viral infections, including influenza virus A and B, parainfluenza, varicella, and severe acute respiratory syndrome coronavirus infections. Abnormal signals on MRI consisted of hypointense and hyperintense signals on T1-weighted and T2-weighted images, respectively. Restricted diffusion patterns of the involved regions could be detected on diffusion-weighted images^1^ ^-^ ^3^.
